# Construction of a Promising Tumor-Infiltrating CD8+ T Cells Gene Signature to Improve Prediction of the Prognosis and Immune Response of Uveal Melanoma

**DOI:** 10.3389/fcell.2021.673838

**Published:** 2021-05-28

**Authors:** Yifang Sun, Jian Wu, Yonggang Yuan, Yumin Lu, Ming Luo, Ling Lin, Shengsheng Ma

**Affiliations:** ^1^Department of Ophthalmology, Guangzhou Red Cross Hospital, Jinan University, Guangzhou, China; ^2^Department of Otorhinolaryngology, Head and Neck Surgery, Guangzhou Red Cross Hospital, Jinan University, Guangzhou, China

**Keywords:** CD8+ T cells, immune-related genes, immunotherapy, prognosis, uveal melanoma

## Abstract

**Background:**

CD8+ T cells work as a key effector of adaptive immunity and are closely associated with immune response for killing tumor cells. It is crucial to understand the role of tumor-infiltrating CD8+ T cells in uveal melanoma (UM) to predict the prognosis and response to immunotherapy.

**Materials and Methods:**

Single-cell transcriptomes of UM with immune-related genes were combined to screen the CD8+ T-cell-associated immune-related genes (CDIRGs) for subsequent analysis. Next, a prognostic gene signature referred to tumor-infiltrating CD8+ T cells was constructed and validated in several UM bulk RNA sequencing datasets. The risk score of UM patients was calculated and classified into high- or low-risk subgroup. The prognostic value of risk score was estimated by using multivariate Cox analysis and Kaplan–Meier survival analysis. Moreover, the potential ability of gene signature for predicting immunotherapy response was further explored.

**Results:**

In total, 202 CDIRGs were screened out from the single-cell RNA sequencing of GSE139829. Next, a gene signature containing three CDIRGs (*IFNGR1*, *ANXA6*, and *TANK*) was identified, which was considered as an independent prognostic indicator to robustly predict overall survival (OS) and metastasis-free survival (MFS) of UM. In addition, the UM patients were classified into high- and low-risk subgroups with different clinical characteristics, distinct CD8+ T-cell immune infiltration, and immunotherapy response. Gene set enrichment analysis (GSEA) showed that immune pathways such as allograft rejection, inflammatory response, interferon alpha and gamma response, and antigen processing and presentation were all positively activated in low-risk phenotype.

**Conclusion:**

Our work gives an inspiration to explain the limited response for the current immune checkpoint inhibitors to UM. Besides, we constructed a novel gene signature to predict prognosis and immunotherapy responses, which may be regarded as a promising therapeutic target.

## Introduction

Uveal melanoma (UM) is the most common intraocular malignant tumor in adult, but much rarer than skin cutaneous melanoma (CM). UM often derives from uveal melanocytes and fast metastasis (Patel, 2013). The incidence of UM is one thousandth of 0.06–0.07, and around 50% of UM patients will eventually die from metastases (Singh et al., 2011; Goh et al., 2020). Despite both UM and CM originate from similar cell types, cancer cells in UM are biologically different from CM (Heppt et al., 2017b). For instance, genic mutations such as *TTN*, *NRAS*, and *BRAF* universally appeared in CM and seldom detected in UM, whereas the mutations of *GNA11*, *GNAQ*, and *BAP1* are commonly observed in UM (Van Raamsdonk et al., 2009, 2010; Cancer Genome Atlas Network, 2015; Livingstone et al., 2020). Moreover, compared with CM, UM bears a lower tumor mutational burden and has a tumor-promoting immune microenvironment (Wang et al., 2020).

Up to now, no systemic treatment has been successfully proven to improve the clinical outcomes of metastatic UM. Despite promising immunotherapies, such as anti-CTLA4, anti-PD1, and anti-PDL1, therapies have been successfully used in CM, and limited response rates toward these immune checkpoint inhibitors were usually observed in UM (Hoefsmit et al., 2020; Qin et al., 2020). For example, the latest clinical outcomes manifested that the 5-year overall survival rate of CM for nivolumab plus ipilimumab therapy was 52% (Larkin et al., 2019). However, the response rate of UM to ipilimumab monotherapy was 0–5% and nivolumab monotherapy was 6%. There was even no response observed to a combination of nivolumab and ipilimumab at median progression-free survival of 2.9 months (Alexander et al., 2014; Zimmer et al., 2015; Heppt et al., 2017a). Notably, higher tumor mutational burden is considered to be closely correlated with higher neoantigens, which tumor-specific T cells may recognize easier (Qin et al., 2020). The mutational burden in CM is known to be much higher than UM, which may partly clarify the distinct response toward immune checkpoint inhibitors. In addition, it is also suggested that tumor-infiltrating T cells take a pivotal role in killing tumor cells, and mediate tumor rejection and antitumor immune responses (Reiser and Banerjee, 2016; Saleh et al., 2020).

For progression cancers, tumor-infiltrating T cells are the most preferred immune cell to effectively target cancer. T-cell density has been demonstrated as a favorable prognostic biomarker for patient survival in glioblastoma, colorectal carcinoma, and ovarian carcinoma (Shionoya et al., 2017). However, compared with many other cancers, the high infiltration of tumor-specific T cells in UM indicated a poor prognosis (Wang et al., 2020). Previous studies proved that tumor-infiltrating CD8+ T cell was the dominated immune cell in UM, which was regarded as a poor prognostic indicator (Bartlett et al., 2014). The opposite effect suggested that different CD8+ T-cell subsets or dysfunction of tumor-infiltrating CD8+ T cells may exist in UM immune environment (Tumeh et al., 2014). Therefore, immune gene-associated tumor-infiltrating CD8+ T cells might be an interesting target to identify gene signature that would possibly improve the response of immunotherapy.

In order to comprehensively evaluate the different subgroups of immune cells and identify the CD8+ T-cell type-specific genes in UM, single-cell RNA sequencing dataset deposited in the Tumor Immune Single-Cell Hub (TISCH) website was first explored. Next, combined with much bulk RNA-seq of UM datasets and corresponding clinical information, we constructed a promising tumor-infiltrating CD8+ T-cell gene signature by using multiple machine learning algorithms. This gene signature may be future targets for rescuing the exhausted CD8+ T cells, stimulating immune surveillance as well as enhancing the efficacy of immune checkpoint blockade therapy.

## Materials and Methods

### Estimation of CD8+ T Cells in Cutaneous Melanoma and Uveal Melanoma

In order to explore the association between tumor-infiltrating CD8+ T cells and clinical outcome in cutaneous and uveal melanoma, the Tumor Immune Estimation Resource (TIMER2.0) database^1^ was used to comprehensively analyze immune infiltrates across diverse cancer types by multiple immune deconvolution methods (Li et al., 2020). Besides, TIMER2.0 affords the Cox regression and Kaplan–Meier survival analyses to estimate the prognostic value of corresponding immune infiltrates in various cancer types.

### Identification of CD8+ T Cell-Associated Immune-Related Genes in Uveal Melanoma

The 7307 CD8+ T cell type-specific genes in UM (Supplementary Table 1) were obtained from the Tumor Immune Single-Cell Hub (TISCH) website^2^, which is a single-cell RNA-seq database and aims to characterize tumor microenvironment at single-cell resolution (Sun et al., 2021). Next, the cutoff criterion of | log2 FC| ≥ 0.5 and adjusted *p* values < 0.05 were applied to screen the different expressed genes (DEGs) in CD8+ T cells. Moreover, the latest version of immune-related genes was acquired from the ImmPort database^3^. Finally, the overlapped genes of DEGs in CD8+ T cells and immune-related genes were regarded as CD8+ T cell-associated immune-related genes (CDIRGs) for subsequent analysis.

### Uveal Melanoma Dataset Collection and Processing

The bulk RNA sequencing datasets of UM as well as corresponding clinical information were downloaded from the TCGA database^4^. Besides, several UM-related gene expression datasets (accession number: GSE22138 and GSE84976)(Laurent et al., 2011; van Essen et al., 2016) deposited in the Gene Expression Omnibus^5^ were also downloaded for outside validation. Moreover, a previous study treated with CTLA-4 and PD-1 blockade therapy was obtained from published literature to predict immunotherapy response (Roh et al., 2017). The raw gene expression datasets were processed by using the following steps: First, probe IDs were annotated to genes by using the Bioconductor package and the corresponding platform annotation profiles. Next, the genes with missing values >50% of samples were excluded. Finally, the raw matrix data were quantile normalized and log2 transformed.

### Construction of CD8+ T Cell-Related Gene Signature

The association between CDIRGs and the overall survival (OS) time of UM patients in TCGA was analyzed. Univariate Cox regression analysis was performed to identify the survival-related genes (*p* values < 0.05). Next, the variable importance (VIMP) algorithm in random survival forest (RF) was used to select the importance of candidate genes, then the multivariate Cox regression method was performed to construct a risk score model with selected CDIRGs. The risk score was calculated as follows: Risk score = $\sum_{i=1}^{\text{N}}\left(c\,o\,e\,f_{i}\times e\,x\,p\,r_{i}\right)$, in which N is the number of genes selected by RF, expri is the expression value, and coefi is the coefficient of genes. Furthermore, the Kaplan–Meier tests were applied to the multiple gene combination signatures, and log-rank *p* values were calculated, which were further used to compare different gene combinations and eventually screened the best gene signature (Sui et al., 2019). Receiver operating characteristic (ROC) analysis for 3- and 5-year OS or metastasis-free survival (MFS) was performed, and area under the curve (AUC) was calculated to assess the sensitivity and specificity of the gene signature. Besides, to test the robustness of the result, this CDIRG gene signature was further verified in the GES22138 and GSE84976 datasets.

### Subgroup Analysis

To evaluate the relationship between risk score distribution and clinical features, the subgroup analyses were separately performed for different types of UM clinical variables including age, stage, histological type, chromosome 3 status, metastasis, and vital status. Besides, in order to evaluate the prognostic value, multivariate Cox regression analysis was performed to determine whether the risk score had a prognostic value independent of other clinical variables.

### Pathway Enrichment Analysis

In order to explore the different signaling pathways between the low- and high-risk groups, the gene set enrichment analysis (GSEA) was conducted. First, the differential analysis of all genes between low- and high-risk groups was generated, and these genes were ordered by the value of log2 fold change. Then gene set databases including cancer Hallmarks (h.all.v7.0.symbols) and Kyoto encyclopedia of genes and genomes (c2.cp.kegg.v7.2.symbols) were used to investigate the signaling pathways correlated with different subgroups of UM. Significance pathway was set at FDR ≤ 0.1 and *p*-value ≤ 0.05, and the top five pathways considered as the most significant are illustrated in the figures.

### Potential Indicator for Immunotherapy Response

To assess the possible ability of risk score for prediction of immunotherapy response, the correlation between the risk score and immune checkpoint genes such as PD-1, CTLA-4, and LAG3 was explored. Most importantly, the immunotherapy response molecular marker—immunophenoscore was also included in our research, which is a well-established predictor of response to checkpoint blockade in melanoma (Charoentong et al., 2017). Next, to investigate the associations between risk score and immune microenvironment, the “CIBERSORT” algorithm was applied to calculate the proportions of immune cells. Then correlation and subgroup analyses between the risk score and these immune cells were conducted. Finally, the tumor immune dysfunction and exclusion (TIDE) algorithm was used to predict clinical response to immune-checkpoint inhibitors, and subclass mapping (SubMap) was performed to compare the expression similarity between the subgroup (high/low risk score) and the melanoma patients with different anti-PD-1 and anti-CTLA-4 therapy responses to predict the efficacy of immunotherapy in UM patients.

### Statistical Analysis

All statistical analyses were conducted by using the R software (v.3.6.0). RF algorithm was calculated by the “randomForestSRC” package (Nasejje et al., 2017). The Kaplan–Meier test and ROC analysis were applied by using the “survival” and “survivalROC” packages (Therneau and Li, 1999; Huang et al., 2020). The best cutoff values were computed by using the “survminer” package (Zeng et al., 2019). The CIBERSORT method was estimated by the “CIBERSORT” package (Newman et al., 2015). GSEA was performed by “clusterProfiler” package (Yu et al., 2012). The correlation analysis was calculated by Spearman test. For comparisons of two groups and more than two groups, unpaired test and one-way ANOVA analysis were used, respectively. Univariate and multivariate Cox regression was used to evaluate the relevant prognostic factors. The hazard ratios (HR) and 95% confidence intervals (95% CI) of the prognostic factors were calculated. *P* < 0.05 was regarded as statistically significant in all statistical tests.

## Results

### Opposite Outcome for CD8+ T Cells in Cutaneous Melanoma and Uveal Melanoma

**FIGURE 1:**
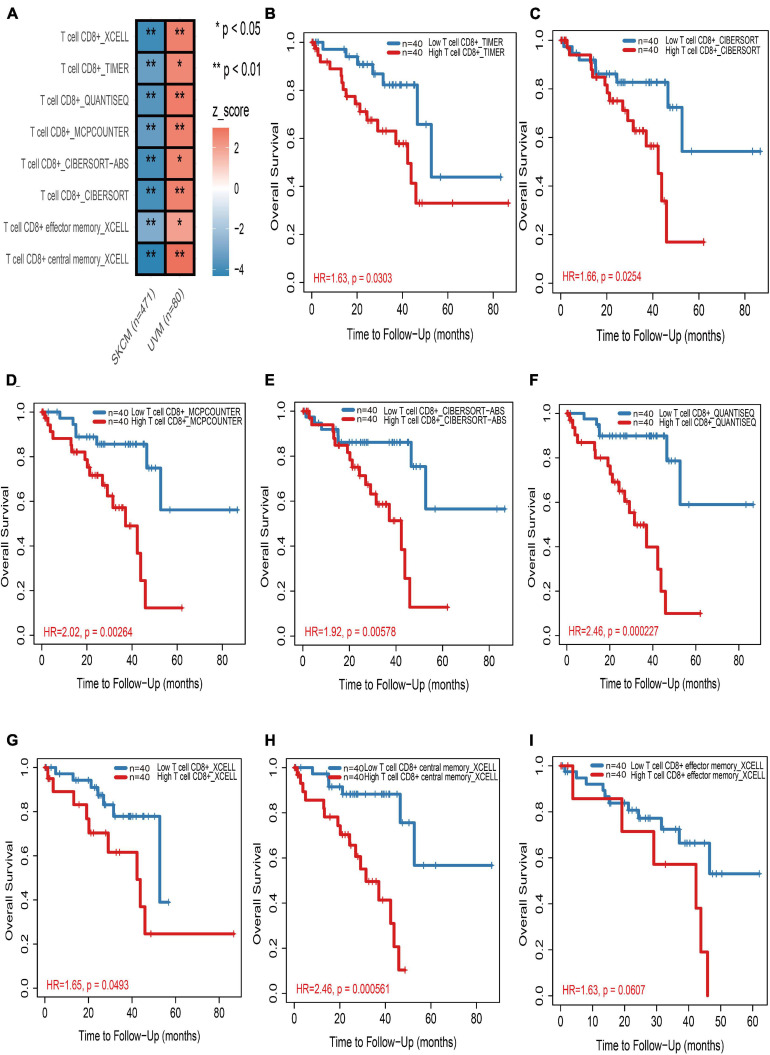
The prognostic value of CD8+ T cells in cutaneous and uveal melanoma (UM). **(A)** Heatmap of multivariable Cox proportional hazard model for CD8+ T cells in cutaneous and UM. Z-score > 0 means increased risk; Z-score < 0 means decreased risk. **(B–I)** Kaplan–Meier survival analysis of CD8+ T cells in UM by TIMER **(B)**, CIBERSORT **(C)**, MCPCOUNTER **(D)**, CIBERSORT-ABS **(E)**, QUANTISEQ **(F)**, and XCELL **(G–I)** methods, respectively. *p < 0.05; **p < 0.01.

In the TIMER2.0 website, multiple immune deconvolution methods including “XCELL” (Aran et al., 2017), “TIMER” (Li et al., 2016), “QUANTISEQ” (Finotello et al., 2019), “MCPCOUNTER” (Becht et al., 2016), “CIBERSORT-ABS,” and “CIBERSORT” (Newman et al., 2015) were used to estimate immune infiltrates in cutaneous and UM. Through univariable Cox proportional hazard model, we astonishingly found that tumor-infiltrating CD8+ T cells work as a protective factor for cutaneous melanoma patients, whereas the increase in tumor infiltration of CD8+ T cells will risk UM patients (Figure 1A and Supplementary Table 2). Kaplan–Meier curves also showed that the high tumor-infiltrating CD8+ T cell subgroup have a significant shorter survival time than the low tumor-infiltrating CD8+ T cell subgroup in UM regardless of which kind of deconvolution method (Figures 1B–I).

### Identification of CDIRGs Based on Single-Cell RNA-Seq

**FIGURE 2:**
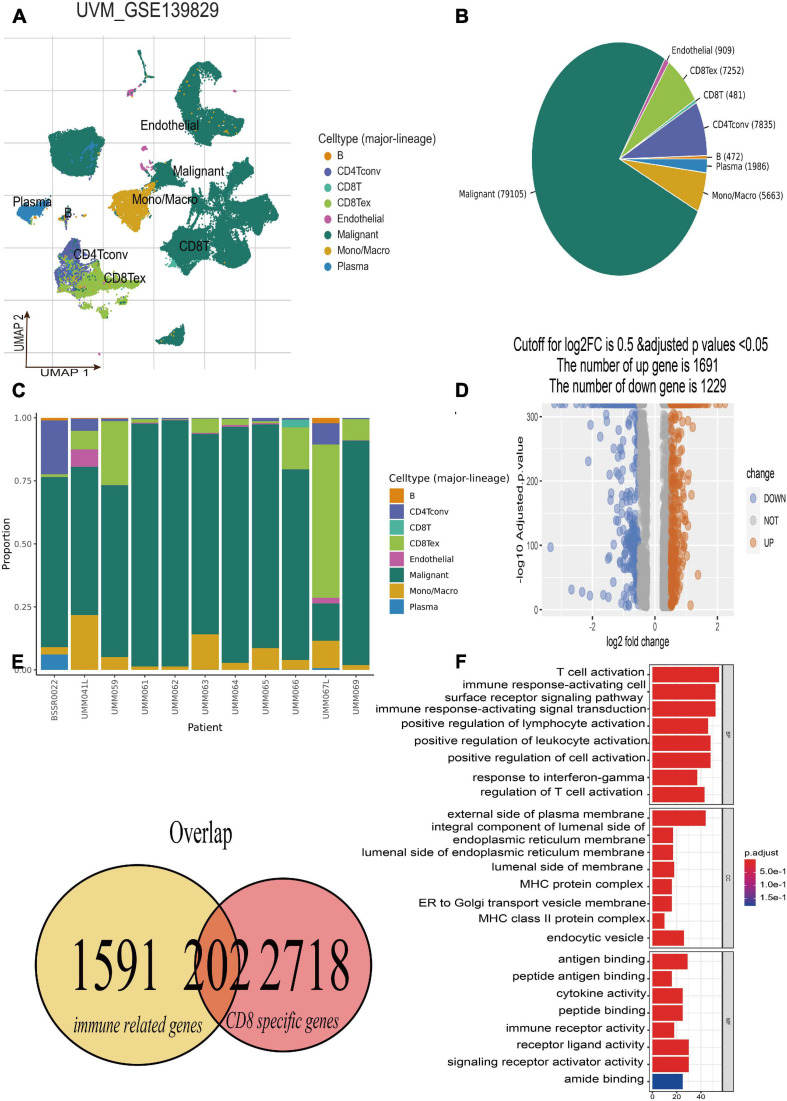
Identification of CD8+ T-cell-associated immune-related genes (CDIRGs). **(A)** The landscape of UM single-cell samples; annotated UMAP plot identified a total of eight different cell types including B cells, CD4+ T cells, CD8+ T and T exhausted cells, endothelial, malignant, mono/macrophage, and plasma. **(B)** The pie plot of eight different cell types. Apart from malignant cells, CD8+ T exhausted cells take a larger component. **(C)** The bar plot for proportion of eight different cell types. **(D)** The volcano plot of the different expressed genes (DEGs) in CD8+ T cells. **(E)** The overlapped CDIRGs of DEGs and immune-related genes. **(F)** Gene Ontology (GO) enrichment analysis of the CDIRGs.

The single-cell RNA-seq of GSE139829 was well processed and deposited in the TISCH website (Durante et al., 2020), which contains 103,703 tumors and non-neoplastic cells from three metastatic and eight primary UM tumors. By applying UMAP algorithms, these mixed cells can be definitely clustered and annotated into eight cell types including B cells, CD4+ T cells, CD8+ T and T exhausted cells, endothelial, malignant, mono/macrophage, and plasma (Figure 2A). The pie plot showed that the number of CD8+ T cells was the main component for UM tumor immune environment (Figure 2B), and the bar plot manifested that the CD8+ T cells take a large proportion for each patient (Figure 2C), respectively. Therefore, the CD8+ T cell-type-specific marker genes were obtained for further analysis. Afterward, according to the selected criterion, 2,920 DEGs were screened out in the GSE139829 dataset, where 1,691 genes were upregulated, and 1,229 genes were downregulated (Figure 2D). Moreover, 1,793 immune-related genes were downloaded from the ImmPort database. Finally, 202 CDIRGs were acquired from the overlapped plot (Figure 2E). The gene ontology (GO) enrichment analysis revealed that these CDIRGs were significantly enriched in T-cell activation, positive regulation of lymphocyte activation, immune response-activating cell surface receptor signaling pathway, MHC protein complex, antigen binding, immune receptor activity, and so on (Figure 2F).

### Construction of CD8+ T-Cells-Related Gene Signature

**FIGURE 3:**
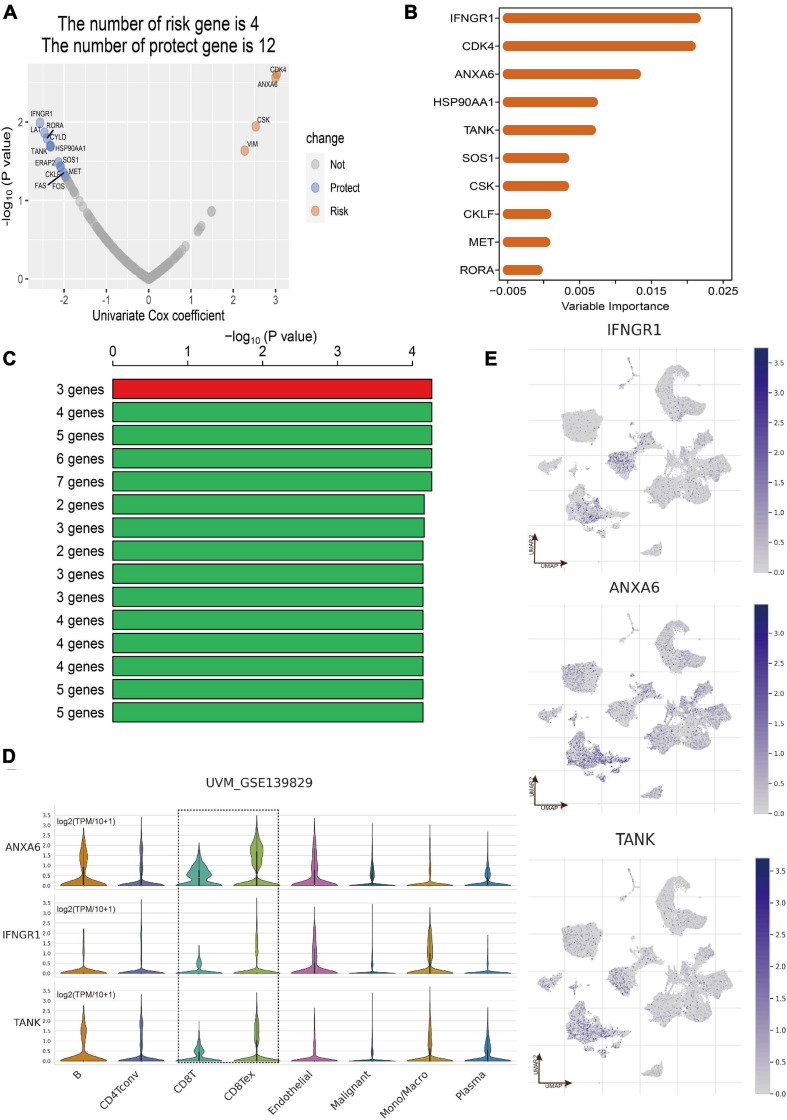
Construction of CD8+ T cell-related prognostic gene signature. **(A)** Volcano plot displayed the CD8+ T-cell-associated immune-related genes (CDIRGs) of the univariate Cox regression analysis. **(B)** Random survival forest analysis screened the most important 10 genes. **(C)** After Kaplan–Meier (KM) analysis, the top 15 gene signatures were sorted according to the log-rank *p* value of KM. A gene signature with three genes was screened out for its big -log10 *p* value and small number of genes. **(D)** Violin plot shows the expression of three genes in different cell types. **(E)** The annotated UMAP plot to check the expression of three genes.

Totally, the RNA sequencing data and clinical information of 171 eligible UM patients were acquired from the three datasets including TCGA of UM (*n* = 80), GSE22138 (*n* = 63), and GSE84976 (*n* = 28). According to the results of overlap between DEGs in CD8+ T cells and immune-related genes, 202 CDIRGs were selected for univariate Cox regression analysis in TCGA dataset and found that a total of 16 CDIRGs was significantly associated with survival of UM patients (*p* < 0.05) (Figure 3A). Next, the top 10 important genes including *IFNGR1*, *CDK4*, *ANXA6*, *HSP90AA1*, *TANK*, *SOS1*, *CSK*, *CKLF*, *MET*, and *RORA* were screened out by the random forest algorithm (Figure 3B). In order to find the optimal gene signature, Kaplan–Meier tests and log-rank *p* values were applied to compare the different gene models. Eventually, the best gene signature contained three genes (*IFNGR1*, *ANXA6*, and *TANK*) with the highest -log10 *P* value selected out (Figure 3C). The violin plot of different cell types in the GSE139829 dataset showed that these three genes had higher expression levels (Figure 3D). The UMAP plots also revealed that these genes were largely expressed in the cluster of CD 8+ T cells (Figure 3E).

**FIGURE 4:**
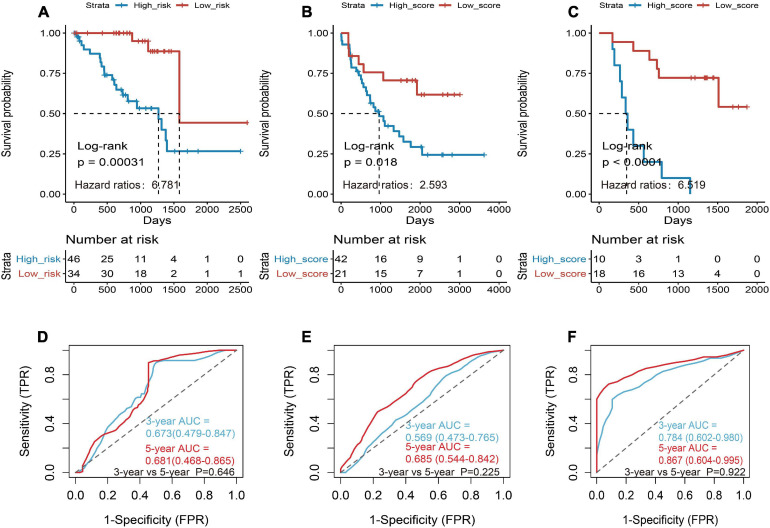
Construction and validation of the prognostic gene signature in UM patients. **(A)** Kaplan–Meier (KM) analysis of risk model for three CD8+ T-cell-associated immune-related gene (CDIRG) signature in TCGA dataset. **(B)** Kaplan–Meier (KM) survival analysis of risk model for three CDIRG gene signatures in the GSE22138 dataset. **(C)** KM survival analysis of risk model for three CDIRG gene signature in the GSE84976 dataset. **(D)** Three and 5 years of the receiver operating characteristic (ROC) curves in TCGA dataset. **(E)** Three and 5 years of ROC curves in the GSE22138 dataset. **(F)** Three and 5 years of ROC curves in the GSE84976 dataset.

Then the three genes were further used to construct a risk score system by applying multivariate Cox analysis in TCGA dataset. According to the formula, a risk score for each patient will be calculated. Afterward, UM patients in TCGA dataset were classified into a high-risk group and a low-risk group by applying the best cutoff value of the risk score. Kaplan–Meier curves showed that patients in the high-risk group have a shorter survival time than the low-risk group with log-rank *p* = 0.00031 and HR = 6.781 (Figure 4A). To estimate the prediction power of gene signature, the ROC curve was drawn, and 3 and 5 years of AUCs were 0.637 (95% CI: 0.479–0.847) and 0.681 (95% CI: 0.468–0.865), respectively (Figure 4D). Besides, verification tests were conducted in GSE22138 and GSE84976 datasets. The GES22138 and GSE84976 datasets were divided into high-risk and low-risk groups accordingly. Kaplan–Meier curves manifested that patients in the high-risk group have a worse prognosis than those in the low-risk group regardless of whether the GSE22138 dataset (log-rank *p* = 0.018 and HR = 2.593) (Figure 4B) or GSE84976 dataset (log-rank *p* < 0.0001 and HR = 6.519) (Figure 4C) was used. The 3 and 5 years of AUCs were 0.569 (95% CI: 0.473–0.765) and 0.685 (95% CI: 0.544–0.842) in the GSE22138 dataset (Figure 4E), and 0.784 (95% CI: 0.602–0.980) and 0.867 (95% CI: 0.604–0.995) (Figure 4F) in the GSE84976 dataset, respectively.

### The Relationship Between Risk Score Distribution and Clinical Features

**FIGURE 5:**
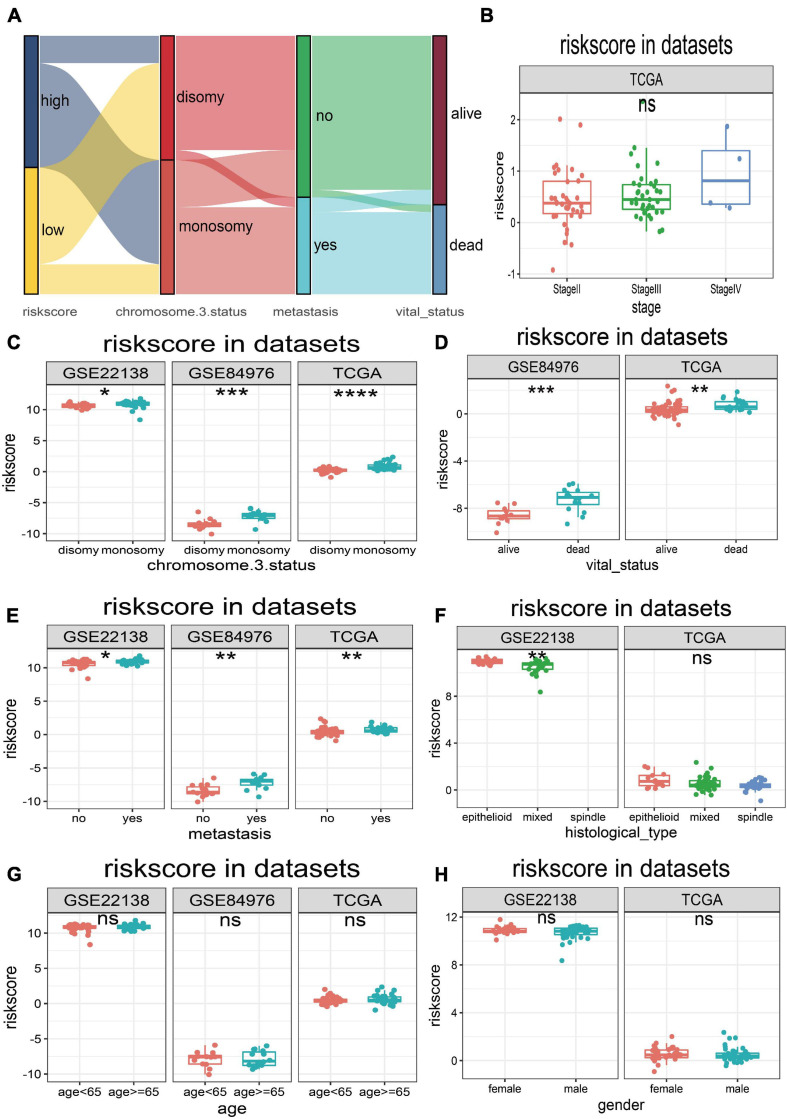
Relationships between risk score and clinical characteristics. **(A)** Sankey plot of risk score distribution in groups with different chromosome 3 status subtype, metastasis, and vital status. **(B)** The risk score distribution of stage in TCGA dataset. **(C)** The risk score distribution of chromosome 3 status in TCGA, GSE22138, and GSE84976 datasets. **(D)** The risk score distribution of vital status in TCGA and GSE84976 datasets. **(E)** The risk score distribution of metastasis in TCGA, GSE22138, and GSE84976 datasets. **(F)** The risk score distribution of histological type in TCGA and GSE22138 datasets. **(G)** The risk score distribution of age in TCGA, GSE22138, and GSE84976 datasets. **(H)** The risk score distribution of sex in TCGA and GSE22138 datasets. *p < 0.05; **p < 0.01; ***p < 0.001; ****p < 0.0001.

**FIGURE 6:**
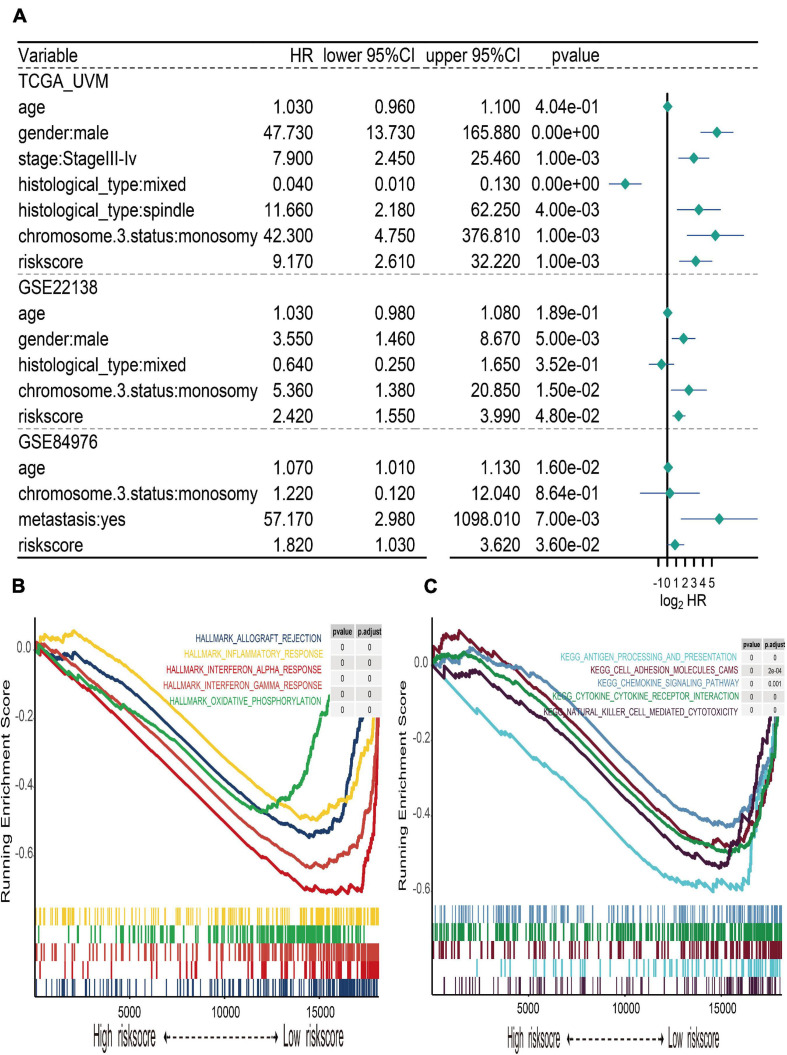
Multivariate Cox regression of risk score and gene set enrichment analysis (GSEA). **(A)** Forest plot of multivariate Cox regression for risk score and clinical characteristics in TCGA, GSE22138, and GSE84976 datasets. **(B)** The top five cancer hallmarks include allograft rejection, inflammatory response, interferon alpha and gamma response, and oxidative phosphorylation, which were enriched in the low-risk group. **(C)** The results of KEGG enrichment included antigen processing and presentation, cell adhesion molecule cams, chemokine signaling pathway, cytokine–cytokine receptor interaction, and natural killer cell-mediated cytotoxicity, which were active in the low-risk group.

The UM patients in TCGA, GSE22138, and GSE84976 datasets were divided into the high- or low-risk score groups by applying the optimal cutoff value. The distribution of patients in the risk score groups, chromosome 3 status, metastasis, and vital status clusters is illustrated in the Sankey plot (Figure 5A). The box plots manifested that chromosome 3 status (Figure 5C), metastasis (Figure 5D), vital status (Figure 5E), and histological type (Figure 5F) were correlated with risk score. Other clinical features, such as age (Figure 5G), gender (Figure 5H), and tumor stage (Figure 5B) had no relationships with risk score. Furthermore, to explore prognostic factors for OS or MFS in multiple datasets, the risk score of gene signature and clinical variables was analyzed by the multivariate Cox regression analyses (Figure 6A). The forest plot revealed that stage, metastasis, chromosome 3 status, histological type, and risk sore were significantly associated with MFS or OS. More importantly, the risk score was significantly correlated with MFS or OS and could be regarded as an independent risk factor in TCGA (HR = 9.170, *P* = 0.001), GSE22138 (HR = 2.420, *P* = 0.048), and GSE84976 (HR = 1.820, *P* = 0.036).

### Gene Set Enrichment Analysis

In order to explore the different hallmark pathways enriched in the high- and low-risk groups, GSEA was performed. According to the ordered pathways enriched in each phenotype, the significant pathways in cancer Hallmarks and KEGG pathway collection were screened out (Supplementary Table 3), and the top five pathways were illustrated in the GSEA plot. The results suggested that hallmarks like allograft rejection, inflammatory response, interferon alpha and gamma response, and oxidative phosphorylation were all enriched in the low-risk group (Figure 6B). The results of KEGG enrichment indicated that the low-risk group was associated with pathways such as antigen processing and presentation, cell adhesion molecule cams, chemokine signaling pathway, cytokine–cytokine receptor interaction, and natural killer cell-mediated cytotoxicity (Figure 6C).

### Potential Indicator for Uveal Melanoma Immunotherapy

**FIGURE 7:**
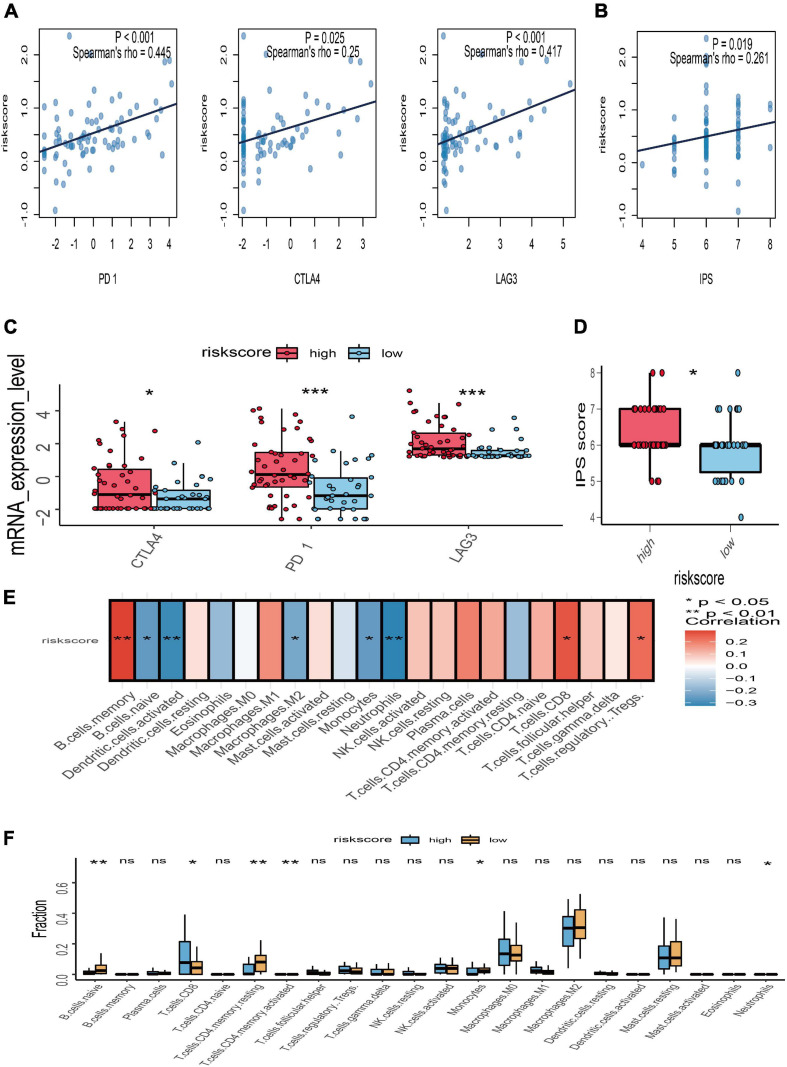
Relationships between risk score with immune checkpoint genes, immunophenoscore (IPS), and immune microenvironment. **(A)** Correlation between the risk score and the expression level of PD-1, CTLA-4, and LAG3. **(B)** Correlation between the risk score and IPS. **(C)** The subgroup analysis of PD-1, CTLA-4, and LAG3 between the high- and low-risk groups. **(D)** The subgroup analysis of IPS between the high- and low-risk groups. **(E)** Heatmap of correlation analysis for the risk score and immune infiltrating cells. **(F)** The subgroup analysis of 22 immune infiltrating cells between the high- and low-risk groups; *p < 0.05; **p < 0.01; ***p < 0.001.

To further explore the potential response for immunotherapy, the association between risk score and the expression level of immune checkpoint genes (PD-1, CTLA-4, and LAG3) was investigated. The correlation analyses manifested that the risk score of gene signature was significantly positively associated with PD-1 (*r* = 0.445 and *p* < 0.001), CTLA-4 (*r* = 0.25 and *p* = 0.025), and LAG3 (*r* = 0.417 and *p* < 0.001) (Figure 7A). The expression value of PD-1, CTLA-4, and LAG3 between the high- and low-risk subgroups was compared; the box plots showed that those in the high-risk group had a significant higher expression level of PD-1, CTLA-4, and LAG3 than those in the low-risk group (Figure 7C). Moreover, immunophenoscore, considered as an effective predictor of immunotherapy, was also positively correlated with risk score (*r* = 0.261 and *p* = 0.019) (Figure 7B). Subgroup analysis indicated that the value of immunophenoscore in the high-risk group was higher than in the low-risk group (Figure 7D). In addition, to explore the association between risk score and immune microenvironment, the CIBERSORT algorithm was first used to calculate 22 immune cells for further investigation of the UM samples (Supplementary Figure 1). Afterward, the correlation analyses between risk score and 22 immune cells suggested that CD8 T cells, regulatory T cells, and B memory cells were positively correlated with risk score, while naïve B cells, activated dendritic cells, M2 macrophages, monocytes, and neutrophils were negatively associated with risk score (Figure 7E). The different analyses of immune infiltration between high- and low-risk score in 22 immune cells indicated that CD8 T cells were highly infiltrated in the high-risk group, and naïve B cells, monocytes, and neutrophils were highly infiltrated in the low-risk group (Figure 7F).

**FIGURE 8:**
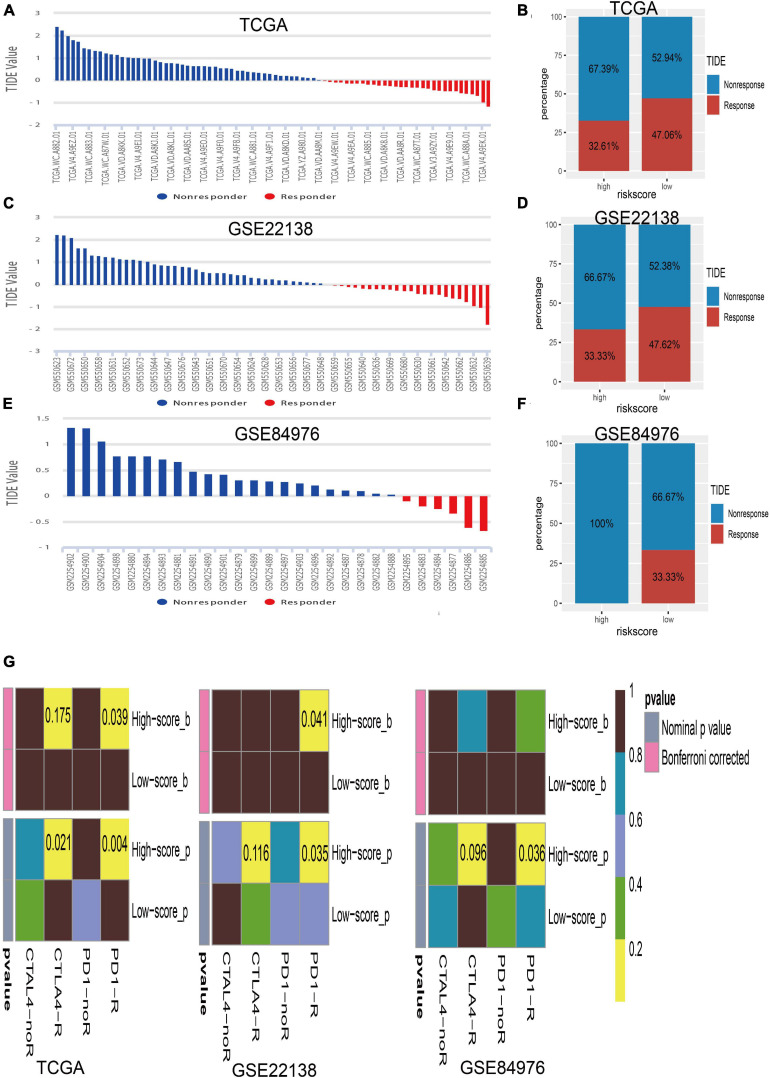
Immunotherapy response of UM. **(A)** Bar plot for the distribution of tumor immune dysfunction and exclusion (TIDE) scores in TCGA dataset. **(B)** The percentage of immunotherapy response between the high- and low-risk groups in TCGA dataset. **(C)** Bar plot for the distribution of TIDE scores in the GSE22138 dataset. **(D)** The percentage of immunotherapy response between the high- and low-risk groups in the GSE22138 dataset. **(E)** Bar plot for the distribution of TIDE scores in the GSE84976 dataset. **(F)** The percentage of immunotherapy response between the high- and low-risk groups in the GSE84976 dataset. **(G)** Submap analysis of different responses for anti-CTLA-4 therapy and anti-PD-1 therapy between the high- and low-risk groups in TCGA, GSE22138, and GSE84976 datasets.

The close associations of the risk score with immune checkpoint genes and tumor immune infiltration prompted us to speculate that the risk score may be used to predict the response for UM immunotherapy. Therefore, we conducted the TIDE algorithm^6^ (Jiang et al., 2018) to calculate the TIDE score for each sample in TCGA (Figure 8A), GSE22138 (Figure 8C), and GSE84976 (Figure 8E). We surprisingly found that the low-risk score group has a larger percentage of response than the high-risk group whether in TCGA dataset (high/low = 32.61%/47.06%; Figure 8B), GSE22138 (high/low = 33.33%/47.62%; Figure 8D), or GSE84976 (high/low = 0.00%/33.33%; Figure 8F). What is more, we performed subclass mapping to compare the expression profile of the high/low subgroups and another published dataset containing 47 patients with melanoma that responded to immune checkpoint inhibitors (CTLA-4 and PD-1) (Roh et al., 2017). Interestingly, we found that the high-risk group is more promising in responding to anti-PD-1 therapy whether in TCGA, GSE22138, or GSE84976 (Figure 8G), whereas, the patients in the low-risk group are insensitive to anti-CTLA-4 or anti-PD-1therapy.

## Discussion

Currently, cancer immunotherapy, regarded as a promising therapeutic method, is generally used in CM patients. However, unresponsive or limited response rates to immunotherapies are often observed in UM patients (Hoefsmit et al., 2020). As we know, successful application of immune checkpoint blockade in CM greatly depends on the ability of anti-tumor immune response, which largely owes to the density of tumor-infiltrating CD8+ T cells (Tavera et al., 2018). Compared with the skin, the eye is regarded as an immune privileged site, which restrains the secretion of immune-mediated cytokines and limited lymph circulation, further increasing retention of tumor antigens and eventually causing CD8+ T-cell exhaustion for continuous exposure (Niederkorn, 2012; Rossi et al., 2019). Therefore, we first performed multiple immune deconvolution methods to comprehensively analyze the prognostic role of tumor-infiltrating CD8+ T cell in UM and CM. The results manifested that higher infiltration of CD8+ T cells in CM indicated a favorable clinical outcome, while larger numbers of CD8+ T cells will decrease the overall survival of UM patients. It is consistent with previous studies that CD8+ T cell refers to favorable prognosis in CM and predicts poor prognosis in UM (Azimi et al., 2012; Gartrell et al., 2018; Wang et al., 2020). Besides, Luo et al. recently identified several prognostic genes in UM, and almost every gene was correlated with abundance in CD8+ T cell (Luo and Ma, 2020). Hence, it is urgent to explore the adaptive immune response gene signature to improve the effect of tumor-infiltrating CD8+ T cell targeting approaches and the response of immunotherapies in UM.

The general RNA sequencing of tumor tissue cannot be representative of CD8+ T cell genomic signature well in UM. Therefore, in this study, single-cell sequencing of UM was used to explore the tumor immune environment and found that CD8+ T cell were the main component immune cell. Besides, exhausted CD8+ T cells take a larger proposition in each UM patient, which is in accordance with the prior reports that UM patients have a higher ratio of exhausted CD8+ T cells (Durante et al., 2020; Hoefsmit et al., 2020). This phenomenon highlights that an immunosuppressive environment exists in UM and suggests that high infiltration of exhausted CD8+ T cells promotes tumor immune evasion. Next, the main concern behind this study was the potential molecular mechanism of CD8+ T cells that regulates the immune tolerance; thus, we screened the CDIRGs based on previous immune-related genes and CD8+ T-cell-specific genes identified from single-cell RNA-seq. Within the CDIRGs, we found that these genes were positively associated with pathways like immune response-activating signal transduction, MHC complex, and immune receptor activity, which further ensure the validity and reliability of our results.

Furthermore, we constructed a prognostic gene signature, which classified the OS or MFS of UM into high- and low-risk groups. Patients in the high-risk group indicated a poor survival. The prognostic gene signature contained three CDIRGs including IFNGR1, ANXA6, and TANK. Interestingly, all these genes have been proven to be associated with cancer or immune response. For instance, IFN-γ signaling is known as an essential effector molecule for anti-tumor immune response, which must bind the IFN-γ receptor (IFNGR1 or IFNGR2) to modulate the JAK–STAT pathways and affects the immune cell activation (Dunn et al., 2005). Several studies reported that the defect in IFNGR1 will promote cancer cells that are unresponsive to immunotherapy, which finally leads to proliferation of cancer cells (Fu et al., 2011; Gao et al., 2016). Annexin A6 (ANXA6) is a superfamily member of membrane-binding annexin proteins, and it has been reported that the expression level of ANXA6 is closely correlated with various cancers (Qi et al., 2015). Rhea et al., suggested that ANXA6 was the most important component of T cell plasma membrane. The lack of ANXA6 was supposed to disturb T-cell proliferation and affect immune signaling pathways (Cornely et al., 2016). Besides, the TRAF family member-associated NF-κB activator (TANK) is regarded as an inhibitor in the immune response via IL1R/TLR activation (Kawagoe et al., 2009). Wang et al. (2015) also reported that TANK may be considered as a therapeutic target to prevent hyperimmune response and improve cancer therapeutic resistance.

To prove the accuracy of gene signature for prognostic prediction, the associations between CD8+ T cell gene signature and clinical parameters were investigated. The results revealed that the risk score of gene signature was intimately correlated with chromosome 3 status, metastasis, vital status, and histological type. Additionally, the multivariate Cox regression analysis also indicated that the risk score of gene signature could be regarded as an independent prognostic factor in UM. Notably, all evidences indicated that the CD8+ T cell gene signature is well constructed and can accurately predict OS or MFS of UM.

Through GSEA, we found that low-risk phenotype has immune activation. Immune pathways such as allograft rejection, inflammatory response, interferon alpha and gamma response, antigen processing and presentation, and cytokine–cytokine receptor interaction were all positively activated. By CIBERSORT estimation, we also observed that the high-risk group have a higher infiltration of CD8 T cells. Thus, it is easy to understand why low-risk UM patients have a better survival outcome than the high-risk group.

Presently, only a few UM patients are responding to immunotherapies in clinical observations. However, we surprisingly found that the risk score has a significant positive correlation with the expression of PD-1, CTLA-4, LAG3, and immunophenoscore. Hence, it is essential to assess the value of gene signature in predicting immunotherapy responses. Luckily, Jiang et al. (2018) developed TIDE algorithm to help researchers identify patients who may benefit from immune checkpoint inhibitors (ICB) more. Combined with TIDE algorithm analysis, we found that low-risk UM patients with a lower TIDE score are more promising in responding to ICB. Therefore, we convinced that this CD8 T cell-related gene signature is a potential indicator of UM immunotherapy response. However, what kind of immune checkpoint inhibitors are suitable for UM is still unclear. Thus, the subgroups with different risk scores were explored in another published dataset containing 47 patients with melanoma who respond to immune checkpoint inhibitors (anti-PD-1 or anti-CTLA-4) (Lu et al., 2019). We surprisingly found that the low-risk group is promising in response to immune checkpoint inhibitors but is unresponsive to anti-PD-1 or anti-CTLA-4 therapy, whereas the high-risk group is sensitive to anti-PD-1 and anti-CTLA-4 therapy, but has a lower TIDE score. These opposite results prompted us to assume that it is urgent to discover and apply novel immune checkpoint inhibitors in clinical treatment. For example, recent studies showed that LAG-3 is the dominant marker in CD8+ exhausted T cells, rather than PD-1 or CTLA-4 (Danaher et al., 2017). Anti-LAG-3 therapy might rescue the exhausted T cells or in an adjuvant approach in treatment of UM (Puhr and Ilhan-Mutlu, 2019; Durante et al., 2020).

To sum up, our study comprehensively constructed a prognostic and immunotherapy responses-related gene signature by integrative analysis of tumor-infiltrating CD8+ T cells, immune-related genes, and clinical information. Our work gives an inspiration to explain the distinct response for the current immune checkpoint inhibitors between CM and UM. Moreover, the gene signature could classify subsets of UM with different infiltrations of CD8+ T cells and afford potential individual immunotherapy in the future.

## Data Availability Statement

The original contributions presented in the study are included in the article/Supplementary Material, further inquiries can be directed to the corresponding author.

## Author Contributions

YS and JW wrote and prepared the original draft. YY, YL, and ML were in charge of the data curation. LL wrote, reviewed, and edited the article. SM was in charge of the project administration. All authors commented and approved the text.

## Conflict of Interest

The authors declare that the research was conducted in the absence of any commercial or financial relationships that could be construed as a potential conflict of interest.

## Footnotes

1 http://timer.comp-genomics.org/

2 http://tisch.comp-genomics.org

3 https://immport.niaid.nih.gov

4 https://portal.gdc.cancer.gov

5 https://www.ncbi.nlm.nih.gov/geo

6 http://tide.dfci.harvard.edu/

## Supplementary Material

Supplementary Figure 1 The landscape of immune infiltration between high and low risk groups.

Supplementary Table 1 The list of CD8+ T cells type-specific genes in UM.

Supplementary Table 2 Univariate cox regression analysis for tumor-infiltrating CD8+ T cells.

Supplementary Table 3 The pathway analysis of the CD8+ T cells related gene signature.
