# Supplementary figures and images for: Construction of a Promising Tumor-Infiltrating CD8+ T Cells Gene Signature to Improve Prediction of the Prognosis and Immune Response of Uveal Melanoma
Source: Front Cell Dev Biol. 2021 May 28;9:673838. doi: 10.3389/fcell.2021.673838 (PMC8194278; doi:10.3389/fcell.2021.673838)

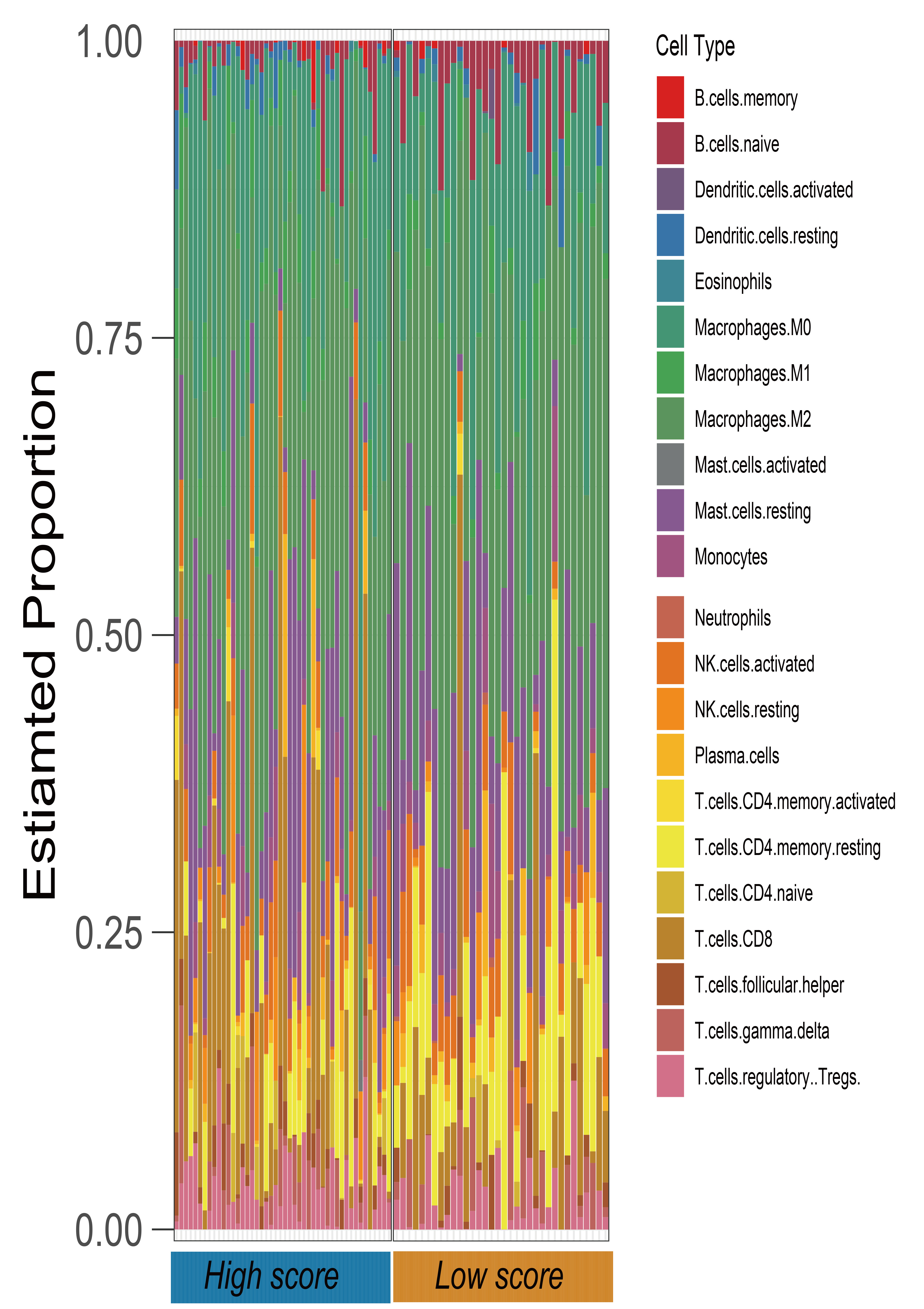

Supplement: Supplementary Figure 1 — The landscape of immune infiltration between high and low risk groups. [file Image_1.JPEG]
